# Nursing engagement in research priorities focused on health systems and services in Latin America countries

**DOI:** 10.1186/s12960-022-00746-9

**Published:** 2022-05-23

**Authors:** Tonda L. Hughes, Maureen George, Ruby Shah, Bruna Moreno Dias, Jennifer E. Dohrn, Silvia Helena De Bortoli Cassiani

**Affiliations:** 1 Columbia University School of Nursing, 560 168th Street, New York, NY 10032 United States of America; 2 Pan American Health Organization, 525 23rd NW, Washington, DC 20037 United States of America

**Keywords:** Research priorities, Universal health coverage, Nursing, Nursing research

## Abstract

**Background:**

A strong nursing research agenda in Latin America is fundamental to universal health coverage. Nursing science can make important contributions to the health of Latin American people through knowledge generation that directly informs nursing practice, professional education, and health policy.

**Methods:**

We used a cross-sectional survey design to assess nursing involvement in health systems and services research in Latin America in five priority areas: *Policies and education related to nursing human resources*; *Structure, organization and dynamics of health systems and services*; S*cience, technology, innovation, and information systems in public health*; *Health policies, governance, and social control*; and *Social studies in the health field*.

**Results:**

Nursing and midwifery participants (*N* = 856) from Latin American countries completed the survey. Respondents who reported conducting research focused primarily on *Policies and Education related to Nursing Human Resources* and *Structure, Organization, and Dynamics of Health Systems and Services.* Across the five priority areas, more nurses reported using research findings and/or being aware of research than conducting research.

**Conclusions:**

Survey results indicate that nursing research in Latin America is currently disproportionately focused on nursing education and practice. More research focused on information technology, nurse’s impact on public health, and the threats posed by nurse migration is needed to better address health needs of Latin American populations.

## Introduction

The Pan American Health Organization (PAHO) is the specialized health agency of the Inter-American System and serves as the Regional Office for the Americas of the World Health Organization (WHO). In consultation with member states, PAHO conducted a study in October 2014 to identify nursing research priorities related to health systems and services in the Americas as set forth in the Strategy for Universal Access to Health and Universal Health Coverage (Resolution CD53/5, Rev. 2) [1]. These priorities were published in 2015 [2] with the expectation that they would guide and support nursing research focused on health systems and services across Latin America. For that study, a systematic literature search was conducted to identify the research priorities and related topics. Initially, a list of 444 topics was generated and evaluated by the PAHO/WHO Collaborating Centers on Nursing and Midwifery, using a consensus method. The topics were then further narrowed to those of greatest relevance by coordinators of graduate programs from 12 Latin American countries and 122 experts in nursing and public health research from 12 countries (Argentina, Bolivia, Brazil, Chile, Colombia, Costa Rica, El Salvador, Mexico, Panama, Peru, Portugal and Uruguay). The final priorities were reviewed and finalized in consultation with Ministries of Health in PAHO member states. These priorities were organized into 14 subcategories within the following six major categories: (1) *Policies and education related to nursing human resources*; (2) *Structure, organization and dynamics of health systems and services*; (3) *Science, technology, innovation, and information systems in public health*; (4) *Financing of health systems and services*; (5) *Health policies, governance, and social control*; and (6) *Social studies in the health field* [2]. Six years later, PAHO in conjunction with the Collaborating Center in the University of Columbia School of Nursing, conducted a survey to determine the extent to which these priorities were guiding current nursing research in Latin America. This paper reports results of the survey.

## Background

Latin America is a region characterized by high inequality and a complex economic, social, and political climate that has direct impact on healthcare access [3]. The many barriers to healthcare access include financial and geographic factors as well as availability and acceptability of health services. These factors affect the provision of comprehensive, efficient, and quality services, especially for vulnerable populations [3]. Additionally, strengthening primary health care services, particularly at the first level of care, in line with the principles of universal health care, remains an urgent priority in the region [4].

In 2009, a PAHO meeting with Latin America and the Caribbean (LAC) stakeholders and leaders focused on limitations in health research in LAC countries and the importance of this research in guiding health system reform. To foster a common agenda, it was recommended that a national policy on research for health be formulated that involved multisectoral participation, articulation, and coordination. Since then, LAC countries have strengthened their national research priorities and are working to develop more consistent approaches to the integration and translation of research that can inform health policies [5]. However, addressing gaps between evidence and policy related to human resources for health and the need to set research priorities and broaden acceptance and use of scientific evidence in informing practice and policy remain important priorities for Latin American countries [6]. To that end, PAHO has been working since 2013 to strengthen and expand the roles and responsibilities of nurses in primary health care systems, including increasing their involvement in research. *Strategic Directions for Nursing in the Region of the Americas*, a PAHO/WHO initiative launched in 2019 [7], highlighted the cross-cutting importance of research and reinforced the need to enhance the translation of research to practice as well as to forge alliances between Ministries of Health and their various stakeholders to support the establishment of a research agenda and set research priorities.

Developing research capacity relies on the crucial support of academic institutions, integration between disciplines and sectors, and the establishment of global partnerships and multilateral initiatives [8]. Nurses represent 56% of the health workforce in the Region of the Americas [9]. More than 1700 Schools of Nursing are listed in the Directory of Nursing Schools of the Region of the Americas. The Latin America Region has more than 1000 nursing schools, and the number of new schools is growing in several countries. Health research in LAC countries is conducted primarily by academic researchers. Although there is a good deal of diversity in research capacity and use of evidence, especially within the scope of health systems and services within and across LAC countries, there remains a great need to expand nursing’s capacity to conduct research and to disseminate and use research findings [10, 11], as well as to build knowledge connections from an intersectoral perspective, with actions that allow integration of the health sector with other sectors, such as education, work and economy.

A strong nursing research agenda is needed to help strengthen health services and systems and promote universal health coverage [12], particularly one that considers topics related to essential services, social determinants of health, and multisectoral involvement. Research with this level of integration, as demonstrated in the COVID-19 pandemic response, will determine the ability of countries to focus their efforts on more effective responses [13].

The current study was designed as a follow-up to the 2014 study [2]. PAHO and the Columbia University School of Nursing in the United States, a PAHO/WHO Collaborating Center in Nursing and Midwifery, conducted a cross-sectional survey of nurses in Latin American countries. The aim of the study was to assess involvement in health systems and services research in Latin American relative to key topics and priorities identified in the earlier study.

## Methods

### Selection and survey topics

Using a consensus approach, a subset of five research priorities and 29 topics considered most relevant to achieving the goal of guiding and supporting nursing research on health systems and services in Latin America was selected from among the 276 topics identified in the 2014 study [2]. These included nine topics focused on Policies and Education related to Nursing Human Resources items, 12 on *Structure, Organization, and Dynamics of Health Systems and Services* items, three on *Science, Technology, Innovation, and Information Systems in Public Health*, three on *Health Policies, Governance, and Social Control* and two on *Social Studies in the Health Field*. No topics from the sixth category, *Financing of Health Systems and Services* were selected for inclusion in the survey.

For each of the 29 topics we asked three questions to assess whether survey respondents: (1) were conducting research related to the topic; (2) were using research findings related to the topic; and/or (3) were aware of research related to the topic. The survey was developed in English, translated into Spanish and Portuguese, and then back translated by native speaking authors. The surveys were anonymous, but captured key sociodemographic and professional characteristics of respondents. Qualtrics, a web-based data collection tool, was used to build and conduct the survey.

### Participant recruitment

Deans, faculty and graduate students at schools of nursing in Latin America with graduate programs, as well as Latin American nurse researchers outside of academia were targeted for recruitment via the PAHO—EnfAmericas listserv, as well as a contact list from the Directory of Nursing Schools of the Region of the Americas (https://www.observatoriorh.org/es/direnf). The e-mail invitation described the purpose of the study and those who received the email were encouraged to share the link with other nurses in Latin America, particularly those engaged in research. E-mail invitations and instructions excluded retired and nonpracticing nurses and midwives as well as editors of nursing journals.

### Survey implementation and dissemination

Email messages in English, Spanish, and Portuguese announcing the study were sent in February 2021, with links to the survey. Eight weeks after the survey opened, email messages announcing the end of the data collection were sent encouraging those who had not yet participated to complete the surveys. The survey was closed in May 2021.

### Ethics review

The Columbia University Institutional Review Board reviewed the survey materials and determined that the project did not constitute human subjects research according to the U.S. federal guidelines (IRB protocol #AAAS8709). The project then was submitted to and approved by PAHO.

## Results

As shown in Table 1, the majority (85.8%) of the 856 nursing and midwifery respondents were female. The largest group of respondents (32.7%) had masters degrees; 29.4% had a Ph.D., DNS, EdD or other doctoral degree; and 14.9% had a bachelor’s degree. Although Latin America does not offer associate degrees in nursing, 20 (2.3%) respondents indicated this as their highest level of education. Based on information entered in the “other, please specify” option, we believe that some respondents misinterpreted the education question. Many of the responses written in for the “other, please specify” option related to positions (associate professor, professor, postdoctoral), student status, or completion of specialty courses.

**Table 1:** Characteristics of nursing and midwifery expert participants

Characteristics	Total (*n*)	Total (%)
Sex		
Female	734	85.8
Male	122	14.3
Age (years)		
18–24	13	1.5
25–34	176	20.4
35–44	236	27.4
45–54	196	22.8
55–64	178	20.7
65+	62	7.2
Education (in nursing)		
Associate degree	20	2.3
Bachelor’s degree	128	14.9
Master’s degree	281	32.7
Ph.D., EdD, other doctoral degree	253	29.4
Other	178	20.7
Employment		
Full time	669	59.5
Part time	102	9.0
Unemployed, actively seeking work	11	1.0
Unemployed/ineligible for work	2	0.2
Retired	22	2.0
Student (doctoral or master’s)	318	28.2
Care setting		
Hospital	205	26.7
General clinic/specialty clinic	49	6.4
Industry (e.g., pharmaceuticals)	1	0.1
Educational institution	411	53.5
Home care	12	1.6
Community care	11	1.4
Public health	80	10.4
Time employed (years)		
Less than 5	72	8.7
5–10	123	14.9
10–20	238	28.9
More than 20	392	47.5
Current position of participants		
Dean	86	5.2
Faculty (school of nursing *with* a Ph.D. program)	235	14.2
Faculty (school of nursing *without* a Ph.D. program)	388	23.5
Nurse employed in health services (e.g., clinic or hospital)	443	26.8
Nurse employed in conducting nursing research in health service delivery	6	0.4
Other	195	11.8

Affiliated nursing school	Yes (*n*)	Yes (%)
Has a Ph.D. program	424	24.9
Has a master’s program	766	65.3
Produces a peer-reviewed scientific nursing journal	513	44.0

About one-fourth (26.8%) of respondents were employed as a practitioner in health services, 23.5% were faculty in a school of nursing without a Ph.D. or Doctor of Nursing Science (DNS) program, and 14.2% were faculty in a school of nursing with a Ph.D. or DNS program. One-fourth (24.9%) of respondents indicated that they work in a nursing school that has a Ph.D. program; two-thirds (65.3%) work in nursing schools that have a master’s degree program. Nearly one-half (44%) reported that the school where they worked or graduated from publishes a peer-reviewed scientific nursing journal.

As shown in Table 2, respondents represent 20 of the 33 Latin American countries; most were from Brazil (26.2%), Mexico (20.7%), or Colombia (11.5%).

**Table 2:** Number of survey respondents by country

Country	Total *n* (%)
Argentina	60 (7.0%)
Belize	4 (0.5%)
Bolivia	9 (1.1%)
Brazil	225 (26.3%)
Chile	71 (8.3%)
Colombia	98 (11.5%)
Costa Rica	9 (1.1%)
Cuba	2 (0.23%)
Dominican Republic	8 (0.9%)
Ecuador	36 (4.2%)
El Salvador	14 (1.6%)
Guatemala	3 (0.4%)
Honduras	14 (1.6%)
México	178 (20.7%)
Nicaragua	14 (1.6%)
Panamá	13 (1.5%)
Paraguay	13 (1.5%)
Peru	41 (4.8%)
Uruguay	25 (2.9%)
Venezuela	10 (1.2%)
Other	9 (1.1%)

### Nursing research priorities

Table 3 summarizes key results of the survey. The percentage of respondents who reported that they conduct research on the nine topics within the priority *Policies and Education related to Nursing Human Resources* ranged from 6 to 31%. More respondents used research (10–48%) or knew about research (14–55%) related to this priority. Within this priority category, most respondents reported conducting, using or being aware of research related to continuing education, training, curricula and competencies. The lowest responses were for conducting or using research related to the effects of nurse migration.

**Table 3:** Respondents’ engagement in nursing research by topic

Topic	Conduct research on this topic	Use research on this topic	Know about research on this topic
	Yes *n* (%)	Yes *n* (%)	Yes *n* (%)
Policies and education related to nursing human resources			
Q1—Research on factors that influence the supply, demand, and geographical distribution of human resources in nursing	152 (14%)	273 (26%)	452 (43%)
Q2—Research on the extent, causes, and effects of nurse migration in your country	87 (8%)	132 (13%)	267 (26%)
Q3—Actions that could reduce nurse migration issues in your country	64 (6%)	101 (10%)	184 (18%)
Q4—Existing models for regulation of nursing professionals in in your country	141 (14%)	189 (18%)	308 (30%)
Q5—Education and/or training models to improve the abilities and competencies (including cultural competencies) of nurses in primary health care settings	320 (31%)	404 (39%)	507 (14%)
Q6—The impact of public health nurses’ education, training, and competencies on the health-related needs of people	246 (26%)	353 (38%)	453 (48%)
Q7—The impact of including current and up-to-date content in the nursing curriculum	272 (29%)	371 (40%)	457 (50%)
Q8—Importance of continuing education for nursing professionals	293 (31%)	412 (45%)	507 (55%)
Q9—Mechanisms to transform nursing human resources education and training curricula	236 (25%)	346 (37%)	417 (45%)
Structure, organization, and dynamics of health systems and services			
Q10—Involvement of nurses in management and/or leadership of health systems and of public health services	196 (22%)	313 (26%)	449 (51%)
Q11—Identifying nursing competencies that are most highly valued by patients and families	210 (24%)	306 (35%)	369 (42%)
Q12—Identifying nursing interventions that are most effective in individual health and/or in population health	316 (36%)	417 (48%)	482 (55%)
Q13—Challenges and strategies used in the implementation of a nursing care model	240 (27%)	343 (40%)	421 (49%)
Q14—Nursing’s role in inter-professional health care	204 (23%)	315 (36%)	377 (44%)
Q15—Ways to support a culture of safety within the health care setting	215 (25%)	312 (36%)	408 (48%)
Q16—The relationship between continuing education policies, quality of nursing care, and rates of institutional adverse events	160 (19%)	281 (33%)	367 (43%)
Q17—The impact of working conditions on health of the nursing workforce	190 (22%)	294 (35%)	414 (49%)
Q18—The relationship between workers’ health conditions, absenteeism rates, and quality of nursing care	156 (18%)	232 (27%)	344 (41%)
Q19—Facilitators and barriers to adequate working conditions	142 (17%)	225 (27%)	311 (37%)
Q20—Evaluation of work-related satisfaction among nurses and/or other health care providers	166 (20%)	243 (29%)	342 (41%)
Q21—Implementation of humanized, comprehensive nursing practice at health facilities	244 (29%)	356 (42%)	458 (55%)
Science, technology, innovation, and information systems in public			
Q22—Access to and utilization of evidence-based information, data, and practices on nursing practice	234 (28%)	386 (47%)	462 (56%)
Q23—Use of information technologies (e.g., big data; data science) in nursing practice	165 (20%)	263 (32%)	317 (38%)
Q24—Institutional policies to support the production of nursing knowledge and/or technology in primary health care settings	148 (18%)	217 (26%)	276 (34%)
Health policies, governance, and social control			
Q25—The role of organizational management in the performance of nursing professionals in the public health sector	143 (17%)	228 (28%)	301 (36%)
Q26—Application of models of governance (shared, clinical, public, etc.) in primary health care settings	87 (10%)	174 (21%)	236 (29%)
Q27—Nurses’ participation in the decision-making process and/or in public policy development	123 (15%)	225 (28%)	298 (37%)
Social studies in the health field			
Q28—Societal recognition of the work of nurses	113 (14%)	212 (26%)	298 (36%)
Q29—Strategies to promote a positive image of nurses/the nursing profession	126 (15%)	199 (24%)	253 (31%)

Slightly more respondents (17–36%) indicated that they were conducting research related to the *Structure, Organization, and Dynamics of Health Systems and Services* with 26–48% indicating that they use, and 37–55% indicating knowing about, research related to this priority. Of the 12 topics within this priority, the largest proportion of respondents indicated that they conduct research related to “identifying nursing interventions that are most effective in individual health and/or in population health.” Leadership, safety, and quality were topics that respondents knew about, but relatively few conducted or used research related to these topics.

For the research priority *Science, Technology, Innovation, and Information Systems in Public Health*, relatively few respondents (18–28%) reported having conducted research on one of the three topics; however, 26–47% reported that they use research, and 34–56% reported knowing about research, related to this priority. Engagement in, use of, and knowledge about was highest for “access to and utilization of evidence-based information on nursing practice.” Fewer respondents endorsed conducting, using or knowing about research related to the use of information technologies in nursing practice or institutional policies to support the production of nursing knowledge and/or technology in primary health care settings.

Similarly, few respondents (10–17%) reported conducting research related to the three topics within the priority *Health Policies, Governance, and Social Control*. About one-fourth (21–28%) reported using research related to this priority and about one-third (29–36%) reported knowing about research within this priority area. Nearly equal proportions of respondents reported conducting, using or knowing about research related to “the role of organizational management in the performance of nursing professionals in the public sector” and “nurse’s participation the decision-making process or in public policy development.”

The research priority, *Social Studies in the Health Field*, included only two topics. Similar proportions of respondents reported conducting and using research related to “societal recognition of the work of nurses” and “strategies to promote a positive image of nurses/the nursing profession”. Slightly more (36%) knew about research related to societal recognition of nurse’s work.

Results for respondents whose affiliated school had a Ph.D./DNS program and those whose affiliated school did not have a Ph.D./DNS program differed. More respondents affiliated with schools of nursing that have doctoral programs reported having conducted, having used, or being aware of research on some topics and more respondents from schools of nursing without doctoral programs reported these forms of engagement on other topics.

## Discussion

Most respondents reported conducting or using nursing research focused on nursing education (competencies and curricula), nursing roles, and nursing interventions to improve individual and/or population health outcomes. To a large, but lesser extent, respondents reported conducting or using research related to evidence-based nursing practice. Research on health systems’ organization and structure was the topic familiar to most study respondents, whereas conducting research or being aware of nursing research that examines health policies, the impact of nursing on public health, nurse migration, and information technologies were least often reported. These findings suggest that some rebalancing of research is needed, with perhaps less focus on education and roles and more focus on health policy, public health, information technology, and especially nurse migration. If nursing curricula included foundational content about the importance of policy, and if advanced education related to policy were available to nurse leaders who can and should influence policy [11], the profession would no doubt be better prepared to address global health challenges such as social, racial/ethnic and sexual/gender inequalities and health inequities [9]. Moreover, by increasing the number of doctoral programs more nurses would have the specialized knowledge and skills to address social determinants of health, human resources for public health, and innovation in health. However, doctoral programs must have the resources, including qualified faculty, to train professionals who have strong skills in statistics, research methods and critical thinking and are capable of conducting rigorous and socially relevant research [14, 15]. The fact that 36% of respondents reported conducting research to identify effective interventions in improving individual health and/or population health (and about 50% reported that they used or knew about this research) is one of the most encouraging findings from the survey.

It is noteworthy that research on issues related to migration was barely explored among respondents, indicating that this topic is ripe for expansion in the coming years. Understanding nurse migration is of even greater importance given the impact of the COVID-19 pandemic on health systems, including its impact on the nursing workforce. Understanding the labor market; issues related to attractiveness, working conditions and retention of nurses; and the capacity of educational systems to address the shortage of bedside nurses and the importance of the clinical and academic nursing workforce in addressing this, are critically important in Latin America and in most parts of the world [16].

Information and communication technology is fundamental to meeting patient safety and care quality needs [17]. So, although it is encouraging that 32% of respondents reported using research informed by data science, the low rate of conducting research using big data highlights another area in which there is need for more nurse scientists to develop programs of research. If nurses were to harnesses the power of predictive analytics patient outcomes could be improved through the identification of evidence-based care that can prevent or ameliorate health problems before they develop.

Themes such as quality and safety, which most participants indicated knowing about, should be a greater focus in future research. This is particularly important given evidence that the quantity of nurses and the quality of their training has measurable impact on care and patient safety [18]. In addition, the provision of adequate working conditions and the safety of health professionals are also essential to providing safe, high quality patient care [19]. Thus, expanding evidence of nursing’s impact on patient safety and quality (and workforce safety) should be of interest to all stakeholders in the healthcare system.

The need to invest in leadership, governance and decision-making is aligned with the Strategic Directions for Nursing and Midwifery policy priorities, which highlight the need to expand management capabilities and leadership positions for nurses. This requires the ability to use research data for high-impact decision-making and implementation of practices and policies geared to the health needs of the population [20].

Understanding health systems and services is the responsibility of nurses in leadership positions at governmental and health service levels. However, the dearth of nurse researchers limits the production of information related to this priority [9]. Focus on health administration and governance in nursing doctoral programs and investment in nursing leadership positions can help increase the production of evidence and foster a greater number of publications and information about governance generated by nurses.

Recently, the WHO led several initiatives related to the health workforce including designating 2020 as the International Year of the Nurse and Midwife, and 2021 as the International Year of Health and Care Workers with the launch of the Global Strategic Direction for Nursing and Midwifery 2021–2025 (SNDM), in April 2021 [20]. To that end, it is important that more nurses in Latin America have senior leadership roles and responsibilities. To support this goal, the nursing profession needs more highly educated nurses. A study on the relationship between the 2030 Sustainable Development Goals (SDGs) and nursing doctoral research in Latin America emphasized the need for nurses with higher levels of education to address the SDGs and highlighted the uneven distribution of doctoral nursing programs in the region [21]. Specifically, of the 53 doctoral nursing programs in Latin America, 72% of them are in Brazil; research conducted in Brazil accounts for 90% of all published clinical nursing and midwifery research in LAC [22]. To expand doctoral nursing programs in Latin America, in 2017 PAHO conducted a study and developed an action plan [11] and has since been working with universities and representatives of ministries of health and education in the region to increase investments in doctoral nursing education and to encourage greater cooperation and collaboration between universities and between countries.

The survey results also highlight the relationship between the number and quality of nursing journals and the investment in graduate education for nurses. Nursing journals in Latin America are typically produced by schools of nursing that have doctoral programs and these peer-reviewed scientific nursing journals are an important means of disseminating nursing research. Despite the expansion of peer-reviewed scientific nursing journals, the production and dissemination of research findings continues to be concentrated in a few countries. Not surprisingly, Brazil leads Latin America in the number of nursing journals and the production of nursing research largely because it has the greatest number of nursing programs that offer graduate degrees (master and doctoral) for nurses. One of the reasons for Brazil’s success is that graduate programs in the country are supported by the Ministry of Education without cost to students. Other Latin American countries need to consider ways to support graduate nursing education as an important means of improving the health of their populations, such as the expansion of graduate educational programs and through collaborative actions and establishment of partnerships [22].

Unfortunately, a substantial proportion of biomedical journals produced in Latin America are not indexed in any major biomedical literature databases. To address this the Iberoamerican Network of Scientific Publishing in Nursing (RedEdit) was created by PAHO in 2006. This network compiles a list of all nursing journals published in Spanish, both in Spain and Latin America, along with descriptions of their focus and primary content [23]. Another initiative, the Portal of Nursing Journals (REV@ENF), which is part of the Virtual Health Library-Nursing (BVS-Enf), aims to expand and strengthen the dissemination of scientific knowledge by providing free access to articles from a selected collection of scientific journals in nursing [24].

### Limitations

The study has several key limitations that should be considered when evaluating the findings. First, because we wanted to ‘cast the net’ as broadly as possible to understand the breadth and depth of nursing engagement in health systems and services research we used a non-probability, convenience sampling strategy which prohibits generalizability and drawing strong conclusions from the findings. Second, the study was cross-sectional, so it is not possible to determine whether or how much impact initiatives to increase nursing research in Latin America has had. Third, to encourage participation, we opted to make the survey anonymous. This precluded collection of more information about the participants and characteristics associated with engagement in nursing research—as well as the possibility of follow-up. Fourth, based on written in responses to the option “other, please specify, in the question about educational level, it appears that some respondents did not understand the question. Thus, it is possible that other questions may not have been clear to respondents—a factor that may also have led to some bias in the findings. However, because the survey instrument was carefully translated and back translated to ensure that respondents could participate using the language version they felt most comfortable using—and because the survey questions were short and uncomplicated, we believe that bias caused by misunderstanding was not a major concern.

## Conclusions and implications

Nursing research is critically important in providing evidence to guide practice, expand the knowledge base, and guide and inform policies that support better health outcomes. It was disappointing therefore, to discover that a minority of survey respondents were engaged in nursing research. Areas of research engagement reported by respondents disproportionately focused on nursing practice and nursing education. Current gaps, such as the use of information technologies and nurse migration, are particularly important topics that need greater attention. The negative impact on the health of people in Latin America caused by migration of their professional nurses is growing concern, particularly given the impact of the COVID-19 pandemic. Migration of nurses in Latin America has long been recognized as an area in need of nursing research. The need to systematically collect and communicate national and regional data to understand why nurses migrate was formally identified in a meeting of nurse leaders from 15 Latin American nearly two decades ago [25]. Although consensus was reached on the need for more data, and further research on these issues and their impact on healthcare, our findings suggests that limited progress toward this goal has been made.

For all topics included in the survey, awareness of research was more common than conducting or using research. Better prepared faculty, especially those educated at the doctoral level, are needed to generate new knowledge. Doctoral-level nursing programs encourage the conduct and utilization of health-related research, and as such they represent a key component in the advancement of nursing science. The generation of scientific knowledge on the topics covered in this report is one path to the achievement of the Universal Health Coverage and Sustainable Development Goals. It is our sincere hope that results of this study will serve to guide and support nursing research on health systems and services that take into consideration the different needs of each country in this region and will serve to advance Universal Access to Health and Universal Health Coverage.

The topics presented here are relevant to the advancement of nursing and to achieving universal health. However, it is important to emphasize that the identification of priorities for nursing research is a dynamic and continuous process that requires constant review and the active participation of various stakeholders.

## Data Availability

The datasets generated and/or analyzed during the current study are not publicly available due to privacy issues, but are available from the corresponding author on reasonable request.

## Abbreviations

IRB: Institutional Review Board
LAC: Latin America and the Caribbean
PAHO: Pan American Health Organization
SDG: Sustainable Development Goals
SNDM: Global Strategic Direction for Nursing and Midwifery 2021–2025
WHO: World Health Organization

## References

[1] Pan American Health Organization. Strategy for universal access to health and universal health coverage. Washington DC: PAHO; 2014. https://iris.paho.org/handle/10665.2/28276.

[2] Cassiani SHDB, Bassalobre-Garcia A, Reveiz L (2015). Universal access to health and universal health coverage: identification of nursing research priorities in Latin America. Rev Lat Am Enfermagem.

[3] Comisión Económica para América Latina y el Caribe (CEPAL), Organización Panamericana de la Salud (OPS), Comisión Económica para América Latina y el Caribe, Organización Panamericana de la Salud. Salud y economía: una convergencia necesaria para enfrentar el COVID-19 y retomar la senda hacia el desarrollo sostenible en América Latina y el Caribe. Santiago: CEPAL; 2020. p. 27. https://repositorio.cepal.org//handle/11362/45840.

[4] Pan American Health Organization. The essential public health functions in the Americas: a renewal for the 21st century. Conceptual framework and description. Washington, D.C.; 2020. https://iris.paho.org/handle/10665.2/53124.

[5] Ghaffar A, Langlois EV, Rasanathan K, Peterson S, Adedokun L, Tran NT (2017). Strengthening health systems through embedded research. Bull World Health Organ.

[6] World Health Organization. Global strategy on human resources for health: Workforce 2030. Geneva: WHO; 2020. https://www.who.int/publications/i/item/9789241511131.

[7] Pan American Health Organization. Strategic directions for nursing in the region of the Americas. Washington D.C.: PAHO; 2019. https://iris.paho.org/handle/10665.2/50956.

[8] Cancedda C, Binagwaho A, Kerry V (2018). It is time for academic institutions to align their strategies and priorities with the sustainable development goals. BMJ Glob Health.

[9] World Health Organization. State of the world’s nursing report—2020. Geneva: WHO; 2020. https://www.who.int/publications/i/item/9789240003279.

[10] Cassiani SHDB, Wilson LL, Mikael SDSE, Peña LM, Grajales RAZ, McCreary LL (2017). The situation of nursing education in Latin America and the Caribbean towards universal health. Rev Lat Am Enfermagem.

[11] Organización Panamericana de la Salud. Formación doctoral en enfermería en América Latina y el Caribe. Washington, D.C.: OPS; 2017. https://iris.paho.org/handle/10665.2/34312.

[12] Schveitzer MC, Zoboli ELCP, Vieira MMDS (2016). Nursing challenges for universal health coverage: a systematic review. Rev Lat Am Enfermagem.

[13] Swaminathan S, Sheikh K, Marten R, Taylor M, Jhalani M, Chukwujekwu O (2020). Embedded research to advance primary health care. BMJ Glob Health.

[14] Joseph PV, McCauley L, Richmond TS (2021). PhD programs and the advancement of nursing science. J Prof Nurs.

[15] Smaldone A, Larson EL (2021). What PhD competencies should guide the preparation of nurse scientists?. J Prof Nurs.

[16] International Council of Nurses. COVID-19 and the international supply of nurses. Geneva: ICN; 2020. p. 27. https://www.icn.ch/system/files/documents/2020-07/COVID19_internationalsupplyofnurses_Report_FINAL.pdf.

[17] Darvish A, Bahramnezhad F, Keyhanian S, Navidhamidi M (2014). The role of nursing informatics on promoting quality of health care and the need for appropriate education. Glob J Health Sci.

[18] Sloane DM, Smith HL, McHugh MD, Aiken LH (2018). Effect of changes in hospital nursing resources on improvements in patient safety and quality of care. Med Care.

[19] World Health Organization. Charter: health worker safety: a priority for patient safety. Geneva PP—Geneva: World Health Organization; 2020. https://apps.who.int/iris/handle/10665/339287.

[20] World Health Organization. Global strategic directions for nursing and midwifery 2021–2025. Geneva: WHO; 2021. https://apps.who.int/iris/handle/10665/344562.

[21] Mendes IAC, Ventura CAA, Silva ÍR, Gir E, de Almeida EWS, Queiroz AAFLN (2020). Alignment and contribution of nursing doctoral programs to achieve the sustainable development goals. Hum Resour Health.

[22] Iribarren S, Stonbraker S, Larsen B, Santos I, Faria R, Góes FSN (2018). Clinical nursing and midwifery research in Latin American and Caribbean countries: a scoping review. Int J Nurs Pract.

[23] Rede Iberoamericana de Editoração Científica em Enfermagem. RedEdit. https://www.braziliannursing.com.br/rededit/. Accessed 14 Oct 2021.

[24] Biblioteca Virtual em Saúde - Enfermagem. REV@ENF - Portal de Revistas de Enfermagem. http://www.revenf.bvs.br/. Accessed 14 Oct 2021.

[25] Siantz MLDL, Malvárez S (2008). Migration of nurses: a Latin American perspective. Online J Issues Nurs.

